# Extensive loss of translational genes in the structurally dynamic mitochondrial genome of the angiosperm *Silene latifolia*

**DOI:** 10.1186/1471-2148-10-274

**Published:** 2010-09-10

**Authors:** Daniel B Sloan, Andrew J Alverson, Helena Štorchová, Jeffrey D Palmer, Douglas R Taylor

**Affiliations:** 1 Department of Biology, University of Virginia, Charlottesville, VA, USA; 2 Department of Biology, Indiana University, Bloomington, IN, USA; 3 Institute of Experimental Botany, v.v.i, Academy of Sciences of the Czech Republic, Prague 6, Lysolaje, 16502 Czech Republic

## Abstract

**Background:**

Mitochondrial gene loss and functional transfer to the nucleus is an ongoing process in many lineages of plants, resulting in substantial variation across species in mitochondrial gene content. The Caryophyllaceae represents one lineage that has experienced a particularly high rate of mitochondrial gene loss relative to other angiosperms.

**Results:**

In this study, we report the first complete mitochondrial genome sequence from a member of this family, *Silene latifolia*. The genome can be mapped as a 253,413 bp circle, but its structure is complicated by a large repeated region that is present in 6 copies. Active recombination among these copies produces a suite of alternative genome configurations that appear to be at or near "recombinational equilibrium". The genome contains the fewest genes of any angiosperm mitochondrial genome sequenced to date, with intact copies of only 25 of the 41 protein genes inferred to be present in the common ancestor of angiosperms. As observed more broadly in angiosperms, ribosomal proteins have been especially prone to gene loss in the *S. latifolia *lineage. The genome has also experienced a major reduction in tRNA gene content, including loss of functional tRNAs of both native and chloroplast origin. Even assuming expanded wobble-pairing rules, the mitochondrial genome can support translation of only 17 of the 61 sense codons, which code for only 9 of the 20 amino acids. In addition, genes encoding 18S and, especially, 5S rRNA exhibit exceptional sequence divergence relative to other plants. Divergence in one region of 18S rRNA appears to be the result of a gene conversion event, in which recombination with a homologous gene of chloroplast origin led to the complete replacement of a helix in this ribosomal RNA.

**Conclusions:**

These findings suggest a markedly expanded role for nuclear gene products in the translation of mitochondrial genes in *S. latifolia *and raise the possibility of altered selective constraints operating on the mitochondrial translational apparatus in this lineage.

## Background

The mitochondrial genomes of flowering plants exhibit a number of characteristics that distinguish them from the mitochondrial genomes of other eukaryotes [1]. They are large and variable in size with ample non-coding content [2], including substantial amounts of "promiscuous" DNA of nuclear and chloroplast origin [3,4] as well as sequences of horizontal origin acquired from the mitochondrial genomes of other land plants [5,6]. Angiosperm mitochondrial genomes also contain numerous introns, some of which have been split such that the resulting gene fragments must be transcribed separately and then *trans-*spliced together [7]. Gene expression also relies on extensive C-to-U (and sometimes U-to-C) RNA editing, in which substitution of specific pyrimidines in the mRNA sequence restores phylogenetically conserved codons [8]. Plant mitochondrial genomes generally experience some of the slowest documented rates of nucleotide substitution [9,10] but are subject to rapid structural evolution [11]. High frequency intra- and intermolecular recombination among large repeated sequences is the rule, generating a heterogeneous pool of genome configurations within a single individual [12-14]. The size and complexity of plant mitochondrial genomes, especially when compared with animals and fungi, make them powerful models for exploring the forces affecting eukaryotic genome structure and evolution.

**Figure 1:**
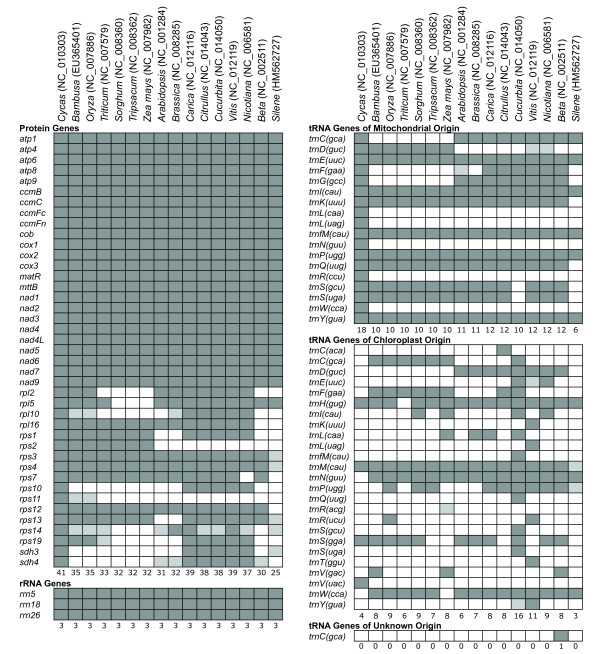
**Gene content in seed plant mitochondrial genomes**. Dark gray boxes indicate the presence of an intact reading frame or folding structure and, therefore, a putatively functional gene, while light gray boxes indicate the presence of a putative pseudogene. The numbers at the bottom of each gene group indicate the total number of intact genes for that species. Note that in some cases the presence of an intact gene sequence may not actually reflect functionality. In particular, for tRNA genes of chloroplast origin, it is possible that transferred sequences still appear intact, but nevertheless, are not functionally expressed in the mitochondrion [37,90]. GenBank accession numbers for each genome are indicated in parentheses.

The genomes of plant mitochondria, like any organelle genome, depend on highly integrated functional coordination with the nucleus. For example, translation of mitochondrially-encoded genes requires a mix of nuclear and mitochondrially encoded components. Plant mitochondrial genomes contain genes for their own rRNA subunits as well as for some of the ribosomal proteins and tRNAs required for translation (Figure 1), but many necessary ribosomal protein and tRNA genes are located in the nuclear genome, so their gene products must be imported into the mitochondrion [15]. The tRNA population within plant mitochondria represents a particularly complex assemblage derived from at least 3 anciently divergent classes of genes [15-17]: 1) "native" tRNAs encoded in the mitochondrial genome and inherited from the α-proteobacterial progenitor of mitochondria, 2) chloroplast-like tRNAs, which are also encoded in the mitochondrial genome but which were acquired by functional gene transfer from the chloroplast genome during land plant evolution, and 3) nuclear-encoded tRNAs imported from the cytosol.

This mixture of tRNA genes is phylogenetically dynamic. Sequenced plant mitochondrial genomes differ in both the number and the identity of tRNA genes that they contain (Figure 1) [4,18]. Likewise, ribosomal protein gene content in the mitochondrial genome is highly variable among plant lineages. The process of mitochondrial gene loss and functional transfer to the nucleus is active and ongoing in plants, and 15 of the 17 protein genes that have been subject to frequent loss across the angiosperm phylogeny encode ribosomal proteins [19-21].

The Caryophyllaceae represents one angiosperm lineage with a relatively high rate of mitochondrial gene loss/transfer. Adams et al. [19] used Southern blots to show that 2 genera from this family (*Dianthus *and *Stellaria*) lack most mitochondrial protein genes outside the core set of 24 genes that are nearly universally conserved throughout angiosperms, and we recently reported that 2 species from a third genus (*Silene*) are similarly reduced in gene content [22]. The genus *Silene *is of particular interest with respect to mitochondrial genome evolution and transmission [23,24]. This large genus exhibits substantial diversity in breeding system, including a high frequency of gynodioecy (mixed populations of hermaphrodites and females), which is often the result of mitochondrial mutations that induce cytoplasmic male sterility [25]. Furthermore, *Silene *species differ markedly in mitochondrial mutation rate [10,26-28] and in the amount of mitochondrial sequence polymorphism that they maintain [26,27,29,30]. Previous analyses of *Silene *mitochondrial genomes, however, have been limited to individual gene sequences.

In this study, we report the complete mitochondrial genome sequence of *Silene latifolia*, which confirms earlier findings of reduced mitochondrial protein gene content in the Caryophyllaceae. We also found a reduction in tRNA gene content that is unprecedented in plants as well as a major increase in the substitution rate for some rRNA genes. In addition, we use paired-end sequence data and Southern blot hybridizations to analyze the complex structural dynamics of this genome, which are driven by a large recombining repeat sequence that is present in 6 copies. These methods could be used more broadly to explore the complex dynamics of mitochondrial genomes in established plant model systems.

## Methods

### Study Species and Plant Material

*Silene latifolia *Poir. (Caryophyllaceae) is a short-lived, herbaceous perennial that is widespread in its native Eurasia [31]. Frequently associated with human disturbance, it is also introduced and invasive in other regions, including North America [32]. Like other members of *Silene *section *Elisanthe*, *S. latifolia *has a dioecious breeding system with XY chromosomal sex determination [25,33].

We grew seeds from a single maternal family in the greenhouse. These seeds were collected by D.R. Sowell from a common garden experiment in Oxford, England, but the maternal plant was derived from seed originally collected on the Apple Orchard Falls Trail in Bedford County, Virginia, USA. A voucher specimen from this family was deposited in the Massey Herbarium at Virginia Polytechnic Institute and State University (D. Sloan #004). Fifteen weeks after the seeds were sown, we harvested 500 g of flowers and fresh green leaves from a total of 550 plants.

### Mitochondrial DNA Extraction, Sequencing, Assembly, and Finishing

We followed previously published protocols for plant organelle DNA extraction [34,35], which yielded approximately 4 μg of mitochondrial DNA. We confirmed the purity of the DNA by digesting a 100 ng sample with PstI and observing a well-defined electrophoretic banding pattern on an agarose gel.

Library construction, cloning, shotgun sequencing, and genome assembly were performed by the Genome Center at Washington University in St. Louis. The genomic DNA was fragmented using a Hydroshear (Digilab; Holliston, MA), end polished, and run on a 0.8% agarose gel. A fraction of that gel corresponding to a 4-6.5 kb size range was excised, purified and ligated into the pSMART vector system (Lucigen; Middleton, WI). After transformation, 2688 subclones were purified, cycle sequenced from both ends with BigDye v3.1 (Applied Biosystems; Foster City, CA), and analyzed on an ABI 3730 capillary sequencer, providing an average of 7× genome sequence coverage.

Shotgun sequence data were assembled with Phrap followed by manual sorting in Consed to resolve misassemblies [36]. Assembly gaps were closed by sequencing subclones with paired-end reads that mapped to the ends of adjacent contigs. Regions with low quality or single read coverage were augmented by PCR and Sanger sequencing of total cellular DNA.

### Genome Annotation

Protein, rRNA, and tRNA genes as well as regions of chloroplast origin were identified using BLAST and tRNAscan-SE as described previously [37]. Regions that were not annotated as belonging to one of these categories were used to search against the NCBI non-redundant nucleotide and protein databases (nt/nr) with BLASTN (r = 5, q = -4, G = 8, E = 6, W = 7, and e = 0.001) and BLASTX (netblast v2.2.19 default parameters except e = 0.001). Perfectly repeated sequences were identified with REPuter [38]. The annotated genome sequence was deposited in GenBank (HM562727).

### Sequence Analysis

Previous studies have shown substantial variation in substitution rates among mitochondrial genes within the genus *Silene *[26,28]. To quantify differences in substitution rate, we analyzed individual protein and rRNA genes in a phylogenetic context with PAML v4.1 [39]. For each gene, we included sequences from 18 seed plant species for which complete mitochondrial genome sequences are available. In these analyses, phylogenetic relationships among the species were constrained according to previous studies [40,41]. For protein genes, branch lengths were estimated in terms of both synonymous and non-synonymous substitutions per site with the program codeml as described previously [28]. For rRNA genes, branch lengths were estimated in terms of substitutions per site with the program baseml. We employed a K80 (Kimura 2-parameter) model of substitution for *rrn5 *(5S rRNA) and an HKY model for *rrn18 *(18S rRNA) and *rrn26 *(26S rRNA). For all 3 genes, we modeled rate variation among sites with a gamma distribution. These substitution models were chosen based on the results of likelihood ratio tests between pairs of competing models. Because the annotated boundaries of rRNA genes differ slightly across species, we trimmed all sequences to the shortest annotated length.

Our analysis revealed a substantial elevation in substitution rate for *rrn5 *in *S. latifolia*. To determine the structural consequences of these substitutions we used the RNAeval program within the Vienna RNA Package v1.8.4 [42] to calculate the free energy of the predicted secondary structure for plant mitochondrial 5S rRNA [43,44]. To test for selection for conservation of secondary structure in *S. latifolia*, we generated 10,000 sequences by randomly placing 16 substitutions (the number observed in *S. latifolia*) into the *Beta vulgaris rrn5 *sequence. *Beta vulgaris *was chosen because it is the most closely related species with an available *rrn5 *sequence, and it appears to have maintained the ancestral sequence of core eudicots. We compared the free energy of the conserved 5S rRNA secondary structure for *S. latifolia *to the distribution of values from the 10,000 simulated sequences to determine whether the *S. latifolia *structure was more highly conserved than expected by chance.

### Southern Blot Hybridizations

We used Southern blots to assess the existence and relative abundance of alternative genome conformations resulting from intramolecular recombination between large repeated sequences. Total cellular DNA was purified from individual fresh leaves using a sorbitol extraction method [45]. Samples were taken from 2 individuals from each of 2 full-sib families. Each of these families was generated by crossing a female from the family used for genome sequencing with a male from an unrelated family. Between 0.5 and 1 μg of genomic DNA was digested with EcoRI (HF enzyme, New England BioLabs), electrophoresed overnight on a 0.9% agarose gel, and transferred to a positively charged nylon membrane (Roche) by capillary blotting. Two probes were generated to target single copy regions flanking large repeated sequences. The probes correspond to genomic positions 140,389-141,463 nt ("left") and 5636-6500 nt ("right") and were generated with the following PCR primers: LeftF1 5'- AGTCTGCCTTTGTCCGACTG; LeftR1 5'- TCCCCTTGGGGTTCTTATCT; RightF2 5'-TCTTTCTTTGCGCTTTCGAT; RightR2 5'-CATTGGCCTTTGCTTCCTT. The probes were labeled with digoxigenin (DIG) using Roche's PCR labeling kit. The genomic blots were hybridized in an EasyHyb buffer (Roche) with the DIG-labeled probe at 42°C overnight, washed at high stringency (0.1× SSC, 65°C), and detected using CDPStar (Roche). An exposure time of 5 to 20 minutes was sufficient to achieve clear bands on ECL film (Kodak). Preliminary data showed that, when amplified directly from genomic DNA, the "right" probe yielded non-specific hybridization, so the PCR fragment was cloned in pGEM T Easy vector (Promega). The resulting plasmid was used as a template to generate the probe with the same PCR primers. The "left" probe was amplified directly from genomic DNA.

## Results

### Genome Size and Organization

**Figure 2:**
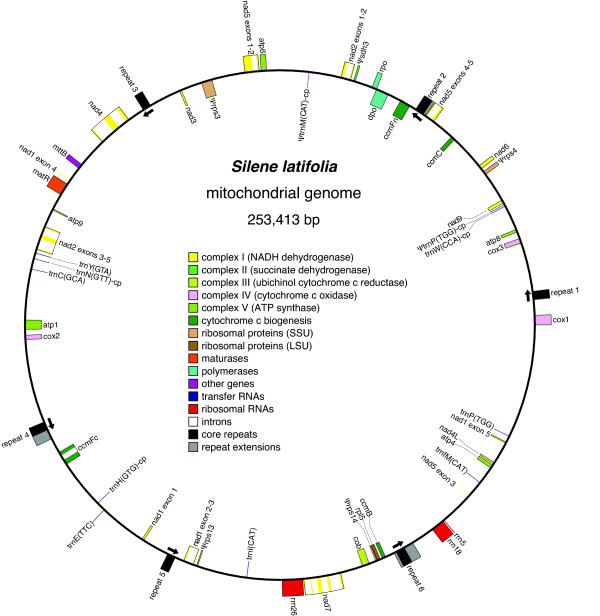
**Mitochondrial genome map**. One of many possible master circle representations of the *Silene latifolia *mitochondrial genome (although this does not necessarily reflect the *in vivo *structure of the genome; see Discussion). Boxes inside and outside the circle correspond to genes on the clockwise and anti-clockwise strand, respectively. Arrows indicate the orientation of repeats as shown in Figure 3. This figure was generated with OGDraw v1.1 [91].

**Figure 3:**
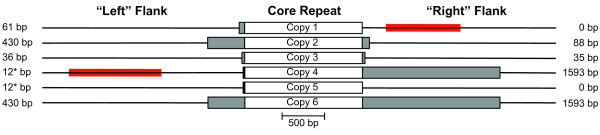
**Structure of large repeated sequences in the *Silene latifolia *mitochondrial genome**. The genome contains a 1362 bp direct repeat present in 6 copies (white boxes). Additional repeat extensions (gray boxes) of varying length are shared by some but not all of the regions that flank the repeat. Shorter repeat extensions are identical in sequence to the initial portions of longer repeat extensions, with the exception of the "left" flanking regions next to repeat copies 4 and 5, which share a short 12 bp sequence (solid black boxes) that is unique relative to the other flanking sequences. Single copy sequences flanking the repeats are shown by thin black lines. The red bars indicate the location of probes used in Southern blot hybridizations (Figure 4). The values on the left and right side indicate the length of the respective repeat extensions. The order of the repeat copies and their flanking sequences corresponds to the genome conformation shown in Figure 2.

The sequenced *S. latifolia *mitochondrial genome can be mapped as a 253,413 bp "master" circle with a total complexity of 244,058 bp if only a single copy of perfectly repeated sequences greater than 100 bp is included (Figure 2). The majority of the repeated sequence in the genome is represented by a 1362 bp "core" repeat sequence that is present in 6 identical copies, all of which are in the same (i.e., direct) orientation relative to each other. Most of the remaining repeated sequence is found in "extensions" of the core repeat. The extensions are identical stretches of sequence between 12 and 1593 bp shared by 2 or more (but not all 6) of the flanking sequences on either the "left" or "right" side of the core repeat (Figure 3). With the exception of this 6-copy repeat and its extensions, the *S. latifolia *mitochondrial genome is relatively devoid of repeated sequences, containing only 2 other repeat families greater than 100 bp (123 bp and 167 bp). Each of these is a 2-copy repeat.

The master circle depicted in Figure 2 represents only one of many possible genome conformations. No single circle is fully consistent with all the sequencing reads because there are numerous paired-end conflicts, i.e., cases where 2 reads from the same subclone map too far apart or in the wrong orientation. With default filtering settings in Consed, these conflicts are exclusively associated with the large 6-copy repeat sequence, suggesting active intra- and intermolecular recombination among repeats [13,14].

**Figure 4:**
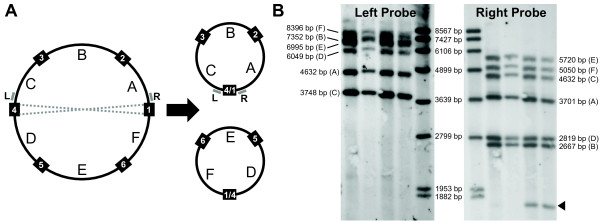
**Recombining repeats in the *Silene latifolia *mitochondrial genome**. (A) A stylized version of the master circle undergoing one of many possible recombination events. The black boxes represent the 6-copy repeat with numbering corresponding to Figure 2. The lettered sections represent intervening single-copy regions. The "left" and "right" probes used in Southern blot hybridizations are indicated with small gray bars and labeled L and R, respectively. The dotted gray lines indicate a crossover event between repeat copies that produces 2 sub-genomic molecules. Given all possible recombination events, each left flanking sequence has the potential to be paired with 6 different right flanking sequences (and vice versa), and therefore, each probe is expected to hybridize to 6 restriction fragments. (B) Southern blot hybridizations with "left" and "right" probes each show 6 strong bands, corresponding to the sizes predicted based on recombination among the 6 large repeats (see Additional File 1 for a more resolved replicate of the "left" probe blot). The left pair of lanes contain DNA samples from one full-sib family, while the right pair contain DNA samples from a second full-sib family. The size standards are indicated by the values between the two blots. The values on either side represent the predicted fragment sizes with the corresponding single-copy flanking sequence noted in parentheses. The black triangle indicates an unexpected 1.8 kb fragment detected in some but not all individuals with the "right" probe.

With 6 copies of the core repeat, there are 36 possible pairs of flanking sequences if all core repeats recombine with each other. Of these, 26 pairs are supported by multiple subclones from our shotgun sequence data. The lack of evidence for the remaining 10 flanking pairs could reflect a reduced frequency or complete absence of these recombination products, but it may also be the result of stochastic sampling and/or cloning bias given our relatively low (7×) sequencing coverage. To distinguish between these possibilities, we first performed (non-quantitative) PCR with all possible pairwise combinations of primers designed for the left and right single-copy regions that flank each core repeat plus its repeat extensions. We detected all 36 possible flanking sequence pairs in DNA extracted from a single leaf (data not shown). We then utilized Southern blots to assess the relative abundance of the various recombination products and confirm that the results from the PCR experiment were not simply an artefact of PCR-mediated recombination [46]. We separately hybridized probes representing one "left" single-copy flanking sequence and one "right" single-copy flanking sequence (Figure 3) to genomic DNA digested with EcoRI. In each case, we detected 6 strong bands, corresponding to the expected sizes of the 6 possible recombination products (Figure 4; Additional File 1). All 6 bands are of similar intensity, suggesting that the alternative conformations of the *S. latifolia *mitochondrial genome exist at relatively equal frequencies. The "right" probe also unexpectedly hybridized to a 1.8 kb fragment, producing a seventh, fainter band that was present in a subset of the individuals (Figure 4). Studies are ongoing to assess the possibility that this seventh band reflects the existence of sublimons and substoichiometric shifting in *S. latifolia *[47,48].

### Gene Content

#### Protein Genes

The *S. latifolia *mitochondrial genome contains intact and putatively functional copies for all 24 of the protein genes that are nearly universally conserved across the large sample of angiosperm mitochondrial genomes examined to date (Figure 1) [19]. In contrast, the genome appears to lack functional copies for most of the 17 other protein genes that were ancestrally present in angiosperm mitochondrial genomes, but which have been subsequently lost, and for the most part, functionally transferred to the nucleus, many times during the course of angiosperm evolution [19,20].

Eleven of these 17 genes have little or no remnant in the genome, while most of the other 6 genes (*rpl5*, *rps3*, *rps4*, *rps13*, *rps14*, and *sdh3*) appear to be pseudogenes. Of this group, only *rpl5 *is fully intact relative to other angiosperms. It is possible that *rps3 *and *rps14 *are functional, but both of these genes show evidence of degeneration. The first exon (75 bp) of *rps3 *has been lost, and the much larger second exon (1773 bp) exhibits a substantial 3' extension before the first in-frame stop codon relative to other angiosperms. The 5' portion of *rps14 *is altered by a frameshift mutation that is corrected after 45 bp by a second frameshift indel. The remaining genes either lack substantial regions that are conserved in other angiosperms (*rps4*) or are truncated by internal stop codons (*rps13 *and *sdh3*). Based on these results, we have identified putatively functional genes and pseudogenes in Figure 1, though a more definitive classification will require detailed analysis of gene expression and function. Regardless, it is apparent that the *S. latifolia *mitochondrial genome has lost a large fraction of the protein genes that were part of the ancestral angiosperm mitochondrial genome.

#### tRNA Genes

The *S. latifolia *mitochondrial genome contains substantially fewer tRNA genes than any angiosperm mitochondrial genome sequenced to date. A search of the genome with BLASTn and tRNAscan-SE identified only 11 tRNA genes, and at least 2 of these (*trnP-cp *and *trnM-cp*) are potential pseudogenes based on the presence of multiple substitutions and insertions in their anticodon loops. A third gene (*trnfM*) shows an elevated substitution rate, but its anticodon and secondary structure appear largely intact (Additional File 2). Five of the 11 genes (including both potential pseudogenes) are of chloroplast origin, representing apparently ancient cpDNA transfers that pre-date the divergence between *Silene *and *Beta*. Collectively, the genes encode a set of tRNAs that, even after including the potential pseudogenes and assuming expanded wobble pairing rules [49,50], can translate only 17 of the 61 sense codons, encoding only 9 of the 20 amino acids (Table 1). By comparison, mitochondrially-encoded tRNAs in *Beta vulgaris *(the most closely related species with a complete mitochondrial genome sequence) can potentially recognize 35 codons, encoding 16 amino acids. Therefore, it is likely that an unusually large fraction of the *S. latifolia *mitochondrial tRNA population is encoded in the nuclear genome and imported from the cytosol.

**Table 1:** Translational capacity of mitochondrially encoded tRNAs in *Silene latifolia*

UUU	Phe	--	UCU	Ser	--	UAU	Tyr	wobble	UGU	Cys	wobble
UUC	Phe	--	UCC	Ser	--	UAC	Tyr	trnY(gua)	UGC	Cys	trnC(gca)
UUA	Leu	--	UCA	Ser	--	UAA	*	--	UGA	*	--
UUG	Leu	--	UCG	Ser	--	UAG	*	--	UGG	Trp	trnW(cca)-cp
CUU	Leu	--	CCU	Pro	wobble	CAU	His	wobble	CGU	Arg	--
CUC	Leu	--	CCC	Pro	wobble	CAC	His	trnH(gug)-cp	CGU	Arg	--
CUA	Leu	--	CCA	Pro	trnP(ugg)^3^	CAA	Gln	--	CGA	Arg	--
CUG	Leu	--	CCG	Pro	wobble	CAG	Gln	--	CGG	Arg	--
AUU	Ile	--	ACU	Thr	--	AAU	Asn	wobble	AGU	Ser	--
AUC	Ile	--	ACC	Thr	--	AAC	Asn	trnN(guu)-cp	AGC	Ser	--
AUA	Ile	trnI(cau)^1^	ACA	Thr	--	AAA	Lys	--	AGA	Arg	--
AUG	Met	trnfM(cau)^2^	ACG	Thr	--	AAG	Lys	--	AGG	Arg	--
GUU	Val	--	GCU	Ala	--	GAU	Asp	--	GGU	Gly	--
GUC	Val	--	GCC	Ala	--	GAC	Asp	--	GGC	Gly	--
GUA	Val	--	GCA	Ala	--	GAA	Glu	trnE(uuc)	GGA	Gly	--
GUG	Val	--	GCG	Ala	--	GAG	Glu	wobble	GGG	Gly	--

^1^The C in the first anticodon position of *trnI(cau) *is assumed to be post-transcriptionally converted to lysidine, which pairs with A.

^2^*trnfM(cau) *transfers formylmethionine and therefore is assumed to recognize only the AUG start codon. The genome also contains a gene with homology to the chloroplast *trnM *gene (which recognizes internal AUG codons), but it has experienced a large expansion in its anticodon loop, making its functionality (and its anticodon) uncertain (Additional File 2).

^3^The genome contains a native *trnP(ugg) *gene as well as a homolog of the chloroplast *trnP(ugg) *gene. The chloroplast-derived copy is likely a pseudogene in *S. latifolia*, as it is believed to be in some other angiosperm mitochondrial genomes [90,93]. Its anticodon loop has experienced multiple substitutions, including one that converts the ancestral UGG anticodon to AGG (Additional File 2).

#### rRNA Genes

Like other angiosperm mitochondrial genomes, the *S. latifolia *genome contains genes encoding 3 ribosomal RNA species (*rrn5*, *rrn18*, and *rrn26*). Two divergent copies of the *rrn5 *gene are present, although one is likely non-functional, exhibiting 3 substantial insertions (7, 9, and 16 bp) and multiple substitutions that greatly reduce the stability of the widely conserved 5S rRNA secondary structure (see below).

### Intron and RNA Editing Content

The *S. latifolia *mitochondrial genome contains a total of 19 group II introns, 6 of which are *trans*-spliced. All 19 introns are found in protein genes, and all but one occur in genes that encode subunits of complex I (NADH dehydrogenase). The *S. latifolia *lineage has lost the second *nad4 *intron and both of the *cox2 *introns found in other angiosperms [51]. It also lacks the group I intron in *cox1*, which has been widely distributed across the angiosperm phylogeny by numerous horizontal transfer events [52]. A previous study identified a total of 287 C-to-U RNA editing sites within the genome's protein genes, which is fewer than typically found in angiosperm mitochondrial genome but substantially more than observed in the rapidly evolving congeners *S. noctiflora *and *S. conica *[22].

### Intergenic Regions

A BLAST search of intergenic regions from the *S. latifolia *mitochondrial genome found that 46.2 kb (23.1%) of this sequence exhibits significant similarity to other land plant mitochondrial genomes (after excluding sequences of clear chloroplast origin). Much of this conserved sequence is directly flanking annotated genes and likely represents regulatory elements, UTRs and *trans*-spliced introns [37]. The genome also contains 2 open reading frames (ORFs) related to the DNA and RNA polymerase genes found on linear mitochondrial plasmids in angiosperms and other eukaryotes [53]. These polymerase genes have also been integrated into the mitochondrial genomes in a number of other angiosperms [54,55].

By searching the complete mitochondrial genome sequence against a collection of diverse chloroplast genomes, we identified a total of 2462 bp of apparent chloroplast origin distributed in 9 fragments ranging in size from 43 to 588 bp. The total chloroplast contribution represents 1.0% of the genome, which is on the low end of the range of approximately 1 to 12% detected in other sequenced angiosperm mitochondrial genomes [37,56]. As found in other angiosperms, the *S. latifolia *mitochondrial genome also contains numerous sequences of apparent nuclear origin, including many regions with homology to (presumably inactivated) angiosperm transposable elements. Nevertheless, based on our search criteria, more than 143.5 kb of intergenic sequence (a full 56.6% of the genome) lacks detectable homology with any DNA or protein sequence in the NCBI nt/nr databases.

### Nucleotide Composition and Codon Usage

The *S. latifolia *mitochondrial genome has a 42.6% GC content, which is slightly below the range of 42.8% to 45.2% observed in other sequenced angiosperm mitochondrial genomes [37,57]. The patterns of codon usage in protein genes (Additional File 3) are very similar to other angiosperm mitochondrial genomes [57], despite the significant changes in tRNA gene content in the *S. latifolia *genome.

### Substitution Rates

**Figure 5:**
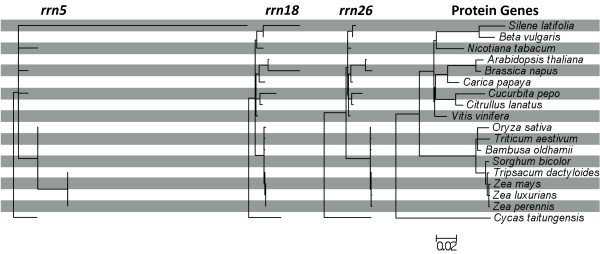
**Phylogenetic analysis of substitution rates in seed plant mitochondrial genomes**. rRNA gene branch lengths are in terms of substitutions per site, while protein gene branch lengths reflect synonymous substitutions per site based on a concatenated dataset of 25 genes present in the mitochondrial genomes of all 18 species. All analyses used a constrained topology.

**Figure 6:**
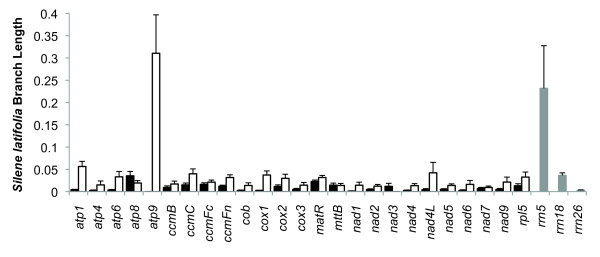
**Substitution rate variation among genes in *Silene latifolia***. Each bar represents the terminal branch length for *S. latifolia *based on a phylogenetic analysis of 18 land plant species with fully sequenced mitochondrial genomes. For protein genes, branch lengths were estimated in terms of non-synonymous substitutions (black bars) or synonymous substitutions (white bars) per site. For rRNA genes, branch lengths were estimated in terms of substitutions per site (gray bars). Error bars represent standard errors, which were calculated as described by Parkinson et al. [79].

Based on a phylogenetic analysis of 18 complete plant mitochondrial genomes, *Silene latifolia *consistently shows higher substitution rates than its sister lineage, *Beta vulgaris *(Figure 5). For the most part, these differences are minor, and the substitution rates in *S. latifolia *are consistent with the low rates that generally characterize plant mitochondrial genomes [10,28]. There are, however, 2 notable outliers with more extreme elevations in substitution rate: the protein gene *atp9 *and the putatively functional copy of the ribosomal rRNA gene *rrn5 *(Figure 6). Elevated substitution rates for *atp9 *have previously been reported throughout *Silene *[28], but this study represents the first analysis of *rrn5 *in the genus.

**Figure 7:**
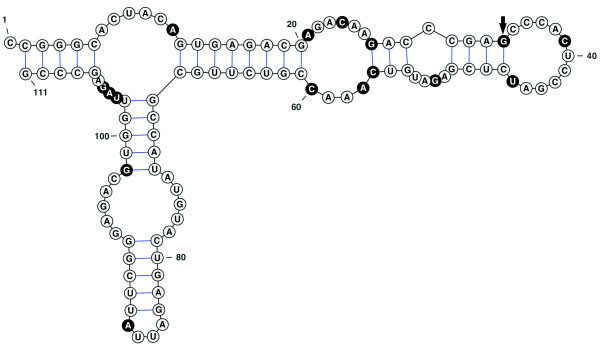
**Predicted secondary structure for *Silene latifolia *5S ribosomal RNA (*rrn5*)**. Sites that have experienced a substitution in the *S. latifolia *lineage are highlighted in black. The black arrow indicates the one predicted change in secondary structure resulting from nucleotide substitution (a novel base pairing between positions 34 and 46). The figure was generated with VARNA v3.6 [92].

Despite their elevated substitution rates, both *atp9 *and *rrn5 *exhibit evidence of purifying selection, suggesting that they are still functionally expressed in the mitochondria. While the observed synonymous substitution rate in *atp9 *is more than 5-fold higher than in any other protein gene in *S. latifolia*, this is the only gene without a single inferred non-synonymous substitution, suggesting strong purifying selection on amino acid sequence (Figure 6; note that *atp9*, at 225 nt in length, is the shortest protein gene in the genome). In the case of the ribosomal rRNA gene *rrn5 *(ca. 111 nt), 13 of the 16 inferred substitutions occur in loops within the conserved secondary structure (Figure 7) [43,44]. Moreover, the 3 substitutions within helices are structurally conservative. Two of those substitutions compensate for each other by altering both bases in a single pairing, resulting in a C:G to G:C change at positions 27:56 (Figure 6). The third substitution found at a conserved helix position (A-to-G at position 98) should still allow for base pairing (G:U instead of A:U). The one predicted change in secondary structure in *S. latifolia *results from a T-to-G substitution at position 34. This position normally represents the first base of the terminal loop on that branch, but the substitution should allow it to pair with C_46 _and extend the preceding helix (Figure 7). As a result, the predicted secondary structure is slightly more stable in *S. latifolia *(*ΔG *= -40.80) than in other angiosperms (e.g., *Beta vulgaris*; *ΔG *= -39.16). A simulation test that randomly placed mutations in *rrn5 *showed that, given the number of substitutions in *S. latifolia*, the conservation of secondary structure is much stronger than expected by chance (*p *< 0.0001). Therefore, it appears that, despite its elevated substitution rate in *S. latifolia*, *rrn5 *is still under selection to maintain folding stability. In contrast, a second *rrn5 *copy in *S. latifolia *is likely a pseudogene, as it contains 3 insertions as well as 3 nucleotide substitutions that disrupt conserved base pairing in helices (*ΔG *= -17.58).

### Gene Conversion Between Mitochondrial and Chloroplast Sequences

**Figure 8:**
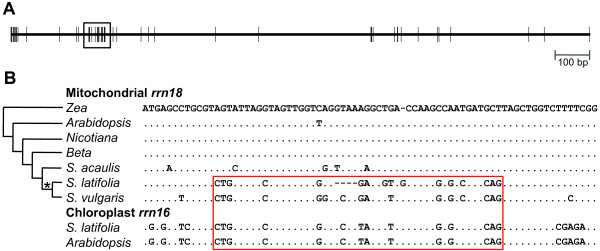
**Gene conversion between mitochondrial and chloroplast small subunit rRNA genes**. (A) The spatial distribution of substitutions (vertical lines) in mitochondrial *rrn18 *that distinguish *Silene latifolia *from *Beta vulgaris *(regions that could not be reliably aligned in a multiple species alignment were excluded). The black box indicates the region shown in detail below. (B) Aligned sequences of angiosperm mitochondrial *rrn18 *and chloroplast *rrn16*. Dots in the alignment indicate sequence identity with the *Zea *reference sequence. The red box shows the minimal extent of the region inferred to have experienced a gene conversion event, which also corresponds to the position of helix 240 in *E. coli *16S rRNA [58]. Analysis of these sequences with GENECONV v1.81a using a mismatch cost of 1 found highly significant evidence for gene conversion in this region (p < 0.0001). The asterisk indicates the inferred phylogenetic timing of that event. Gene sequences were taken from published genomes (see Figure 1) with the exception *S. acaulis *and *S. vulgaris rrn18 *(GenBank EF547249 and HM562728) and *S. latifolia rrn16 *(AB189069).

The distribution of substitutions contributing to the elevated *rrn18 *divergence in *S. latifolia *is noticeably clustered (Figure 8a). One cluster of substitutions is likely the result of a gene conversion event in which a segment of at least 47 bp of *rrn18 *sequence was converted by a homologous chloroplast *rrn16 *gene (Figure 8b). The boundaries of this apparent conversion tract correspond precisely to the beginning and end of helix 240 (domain I) in the secondary structure model for 16S rRNA in *Escherichia coli *[58]. Therefore, the result of the gene conversion appears to have been a clean exchange of the entirety of this helix. The region appears to have been further modified by multiple substitutions and indels since the conversion event. Evidence of this conversion is also present in *S. vulgaris*, but not in *S. acaulis*, indicating that it occurred after the split between the two *Silene *subgenera but before the divergence of the major lineages in subgenus *Behenantha *[28]. We did not find evidence of cpDNA-mediated conversion in any other *Silene *mitochondrial genes, including the rapidly evolving *rrn5 *and *atp9 *genes.

## Discussion

### Mitochondrial Gene Loss

The vast majority of genes in plant mitochondrial genomes can be placed into one of two functional categories: 1) bioenergetics, i.e., oxidative phosphorylation and ATP synthesis (*atp*, *ccm*, *cob*, *cox*, *nad*, and *sdh *genes) and 2) translational machinery (ribosomal protein, rRNA, and tRNA genes). Analysis of the phylogenetic distribution of protein genes across seed plants has clearly shown that ribosomal proteins are subject to more rapid rates of loss than genes involved in bioenergetics [19]. The complete sequence of the *S. latifolia *mitochondrial genome provides the first evidence that mitochondrial tRNA genes, another component of the organelle's translational machinery, can also be lost rapidly and in large numbers in plants. This finding is consistent with broader patterns in eukaryotic evolution, as numerous independent lineages have experienced the loss of most or even all of their mitochondrially-encoded tRNAs [59]. The present study also extends earlier work that found reduced protein gene content in 2 other genera in the Caryophyllaceae [19]. The similar reduction in protein gene content in these 3 taxa suggests that much of the observed protein gene loss probably occurred prior to the diversification of this family, although some degree of parallel loss within the family is also possible.

Protein genes that are lost from mitochondrial genomes can experience a variety of fates. For example, the evolutionary history of eukaryotes has been characterized by a massive physical transfer of genes from the mitochondrial genome to the nucleus. This process is ongoing in plants, and there are a number of well-established cases of such endosymbiotic gene transfer that have occurred since the divergence of angiosperms [60-63]. Losses can also occur when a gene is functionally replaced by an anciently divergent homolog [20,21,64,65], and when a protein or even an entire multi-subunit complex is no longer functionally required (e.g., the loss of the NADH dehydrogenase complex I in apicomplexans and at least 2 yeast lineages [66,67]). In *Silene*, an analysis of the *S. vulgaris *transcriptome (unpublished data) revealed evidence of nuclear copies for at least 9 of the protein genes that appear to have been functionally lost from the *S. latifolia *mitochondrial genome.

To the best of our knowledge, a functional transfer of a mitochondrial tRNA gene to the nucleus has never been documented. Instead, mitochondrial tRNA gene loss is typically offset by importing tRNAs of eukaryotic nuclear origin from the cytosol [17,59]. Therefore, it is likely that *Silene *mitochondria import a greatly expanded set of nuclear tRNAs relative to other plants--a prediction that could be tested by purifying and sequencing *Silene *organelle tRNAs.

In some specific cases, however, more complex evolutionary changes may be required to explain the loss of mitochondrially-encoded tRNAs. For example, in plant mitochondria, the function of tRNA-Gln is dependent on coordinated enzymatic processes. Aminoacyl tRNA synthetases play an essential role in translation by matching tRNAs with their corresponding amino acids, but plant organelles generally lack a Gln tRNA synthetase. Instead, tRNA-Gln is typically aminoacylated by a Glu tRNA synthetase followed by a chemical modification (amidation) to convert Glu to Gln [68].

The gene encoding tRNA-Gln (*trnQ*) is present in all sequenced seed plant mitochondrial genomes with the exception of *S. latifolia*. The loss of the mitochondrially-encoded copy of tRNA-Gln in *S. latifolia *raises several possibilities. First, it is conceivable that aminoacylation and amidation are carried out in the same fashion with an imported cytosolic tRNA-Gln. This may be unlikely, however, because it would require associated changes in tRNA recognition for multiple enzymes. Second, it is possible that, unlike other plants, *S. latifolia *imports the cytosolic Gln tRNA synthetase into its mitochondria, allowing for direct aminoacylation of an imported tRNA-Gln without the use of a Glu intermediate. Finally, it is possible that *S. latifolia *has experienced an unprecedented transfer of a functional tRNA gene (*trnQ*) from the mitochondrial genome to the nucleus, where it is expressed and its product targeted back to the mitochondria. All of these possibilities should be investigated to better understand the mechanisms involved in the co-evolution of organellar and nuclear gene content.

It is intriguing that extensive gene loss in two components of *Silene *mitochondrial translation machinery has been associated with accelerated evolutionary rates in a third component, rRNA genes. This pattern raises the possibility of a correlated reduction in functional constraint across these 3 translational components. A general relaxation of selection on organelle translation has been observed in cases such as the chloroplasts of non-photosynthetic plants where the organelle's functional role has been greatly reduced [69]. However, we have no *a priori *reason to expect relaxed selection on mitochondrial gene expression in *Silene*, and the distribution of substitutions in *rrn5 *suggests that its secondary structure is under strong selection to maintain function. Broader comparative and functional analyses would be of value in assessing the extent to which correlated evolutionary pressures act on these 3 components of mitochondrial translation machinery.

An alternative interpretation of our results is that, rather than being lost, certain genes have been functionally retained in the mitochondrial genome but escaped detection by our annotation methods. For example, cryptic genes could result from accelerated rates of evolution or the proliferation of introns and RNA editing sites [18,70]. Although these explanations are unlikely given the generally slow rate of plant mtDNA sequence evolution and the trend towards a reduced frequency of introns and RNA editing in *Silene *[22], they certainly cannot be ruled out. Likewise, it is possible that some of the gene fragments that we have classified as pseudogenes are functional. Mitochondrial tRNAs often exhibit aberrant or non-canonical secondary structures, making detection of genes and the assessment of functionality more difficult [71,72]. Under any of these scenarios, however, it is still evident that the *S. latifolia *lineage has experienced a period of significant evolutionary change in its mitochondrially-encoded translation machinery.

### Mitochondrial Substitution Rates and Gene Conversion with Chloroplast Genes

Given that the divergence between mitochondria (proteobacteria) and chloroplasts (cyanobacteria) spans billions of years of evolution [73], the notion that gene conversion is occurring between their respective genomes is rather astonishing. Nevertheless, examples of conversion between the mitochondrial *atp1 *and chloroplast *atpA *genes have been documented in multiple angiosperm lineages [74]. The *S. latifolia *mitochondrial genome sequence provides compelling evidence for a similar history of conversion in an rRNA gene. Evidence of recombination between divergent rRNA sequences has also been found in free-living bacteria and archaea [75-77], including one other example of a chimeric proteobacterial/cyanobacterial small subunit rRNA [78].

In all documented cases of apparent conversion between mitochondrial and chloroplast genes, the mitochondrial gene acted as the recipient, which may reflect the propensity of angiosperm mitochondrial genomes to acquire and retain "promiscuous sequences", including those of chloroplast origin. If a conversion event in *Silene *did result from a copy of chloroplast *rrn16 *that had been incorporated into the mitochondrial genome, the promiscuous sequence must have been subsequently lost, because it is no longer present in the *S. latifolia *mitochondrial genome.

The history of gene conversion in *S. latifolia rrn18 *was readily detectable because the conversion tract (47 to 60 bp in length) introduced a distinct cluster of 14 substitutions (although 2 of these appear to have been obscured by subsequent mutations; Figure 8). These changes contributed to an accelerated *rrn18 *substitution rate in *Silene *(Figure 5). Although we did not identify other clusters of substitutions that could be readily explained by gene conversion with homologous chloroplast sequence, it is conceivable that more localized conversion events occurred but escaped detection. It would be difficult if not impossible to distinguish conversion events that introduce only 1 or 2 substitutions from *de novo *point mutations. It has been hypothesized that increases in the frequency of gene conversion with reverse transcribed mitochondrial mRNA ("mutagenic retroprocessing") might explain elevated evolutionary rates in some angiosperm mitochondrial genomes [79]. Given the evidence for gene conversion between mitochondrial and chloroplast genes, the role of DNA-mediated conversion between divergent homologs (or even non-homologous sequences that share small regions of similarity) should be investigated as another potential source of mutational input in plant mitochondrial genomes.

### Repeats, Recombination, and Genome Structure

With rare exception [80], the structure of angiosperm mitochondrial genomes is characterized by the presence of large repeated sequences that facilitate intra-and intermolecular recombination [12,14]. These repeats are generally present in 2 or sometimes 3 copies. In this study, we identified an unprecedented 6-copy family of large, actively recombining repeats in the *S. latifolia *mitochondrial genome. Given a repeat family of this size and recombinational activity, there are 120 different possible conformations for the idealized "master circle", which differ in the precise order of the 6 single-copy regions. The genome structure depicted in Figure 2 represents one of these possible conformations. However, the genome organization is much more complex than any single circular representation for at least 2 reasons. First, a 6-copy family of recombining repeats will potentially generate hundreds of possible subgenomic circles containing anywhere from 1 to 5 repeat loci, as well as a theoretically infinite number of supergenomic circles through multimerization. Second, plant mitochondrial genomes have been shown to exist *in vivo *as a complex assemblage of linear, circular and branched molecules [81,82].

As observed in cases of repeat families with lower copy number [12,83-89], our Southern blot hybridizations confirm the co-existence of multiple alternative genome conformations. The similar intensity of each band (Figure 4) suggests that recombination among the repeats is sufficiently frequent that the many possible pairs of flanking sequences occur at relatively equal levels, a condition defined as "recombinational equilibrium" [13]. Moreover, the repeat copies appear to be completely identical in sequence, providing further evidence for a high rate of homogenization through recombination/gene conversion.

For this study, we utilized Southern blots and *in silico *predictions from a completely sequenced plant mitochondrial genome to provide a semi-quantitative assessment of recombination activity. Extending these methods to other sequenced genomes that differ in the number and size of repeat families could provide valuable comparative data on recombination activity in plant mitochondria. Moreover, the advent of DNA sequencing technologies (e.g., 454 and Illumina) that produce deep sequencing coverage of large span paired-end libraries can provide an opportunity to generate quantitative estimates of the relative abundance of alternative genome conformations.

## Conclusions

Overall, the patterns of gene loss and divergence in the *S. latifolia *mitochondrial genome suggest a markedly expanded role for nuclear gene products in the translation of mitochondrial genes. Furthermore, the novel, recombinationally active repeat structure of this genome represents a complex elaboration of one of the long list of unique features that distinguish plant mitochondrial genomes. With ongoing efforts to sequence the mitochondrial genomes of other *Silene *species that differ profoundly in mitochondrial mutation rates and breeding system, the *S. latifolia *mitochondrial genome should provide a valuable comparative model for investigating the evolutionary forces that shape genome organization.

## Authors' contributions

DBS planned the study, extracted DNA, performed genome finishing and most of the data analysis, and drafted the manuscript. AJA extracted DNA and helped plan the study, analyze the data and draft the manuscript. HŠ performed the Southern blot analysis and helped draft the manuscript. JDP and DRT helped plan the study, analyze the data and draft the manuscript. All authors read and approved the final manuscript.

## Supplementary Material

Additional file 1 **Southern blot hybridizations**. Click here for file

Additional file 2 **Predicted secondary structures of mitochondrially-encoded tRNAs**. Click here for file

Additional file 3 **Summary of codon usage**. Click here for file
