# Supplementary material for: Extensive loss of translational genes in the structurally dynamic mitochondrial genome of the angiosperm Silene latifolia
Source: BMC Evol Biol. 2010 Sep 10;10:274. doi: 10.1186/1471-2148-10-274 (PMC2942850; doi:10.1186/1471-2148-10-274)
Supplement: Additional file 1 — Southern blot hybridizations. [file 1471-2148-10-274-S1.PDF]

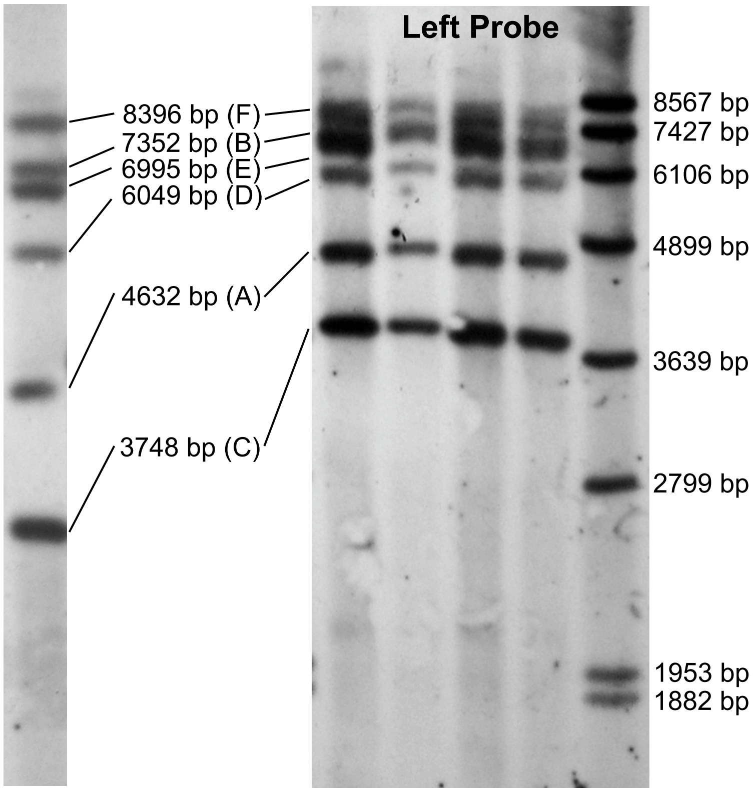

Additional File 1. Resolving higher molecular weight bands in Southern blots. The Southern blot hybridization on the right is duplicated from Figure 4 (see main text for details). The single lane on the left is from a replicate blot (same restriction enzyme and probe) that was derived from a longer gel electrophoresis run to improve separation between large, co-migrating fragments. The DNA sample used for the replicate blot corresponds to the leftmost lane of the original blot.
