# Supplementary material for: Extensive loss of translational genes in the structurally dynamic mitochondrial genome of the angiosperm Silene latifolia
Source: BMC Evol Biol. 2010 Sep 10;10:274. doi: 10.1186/1471-2148-10-274 (PMC2942850; doi:10.1186/1471-2148-10-274)
Supplement: Additional file 2 — Predicted secondary structures of mitochondrially-encoded tRNAs. [file 1471-2148-10-274-S2.PDF]

Native tRNAs

trnC-GCA

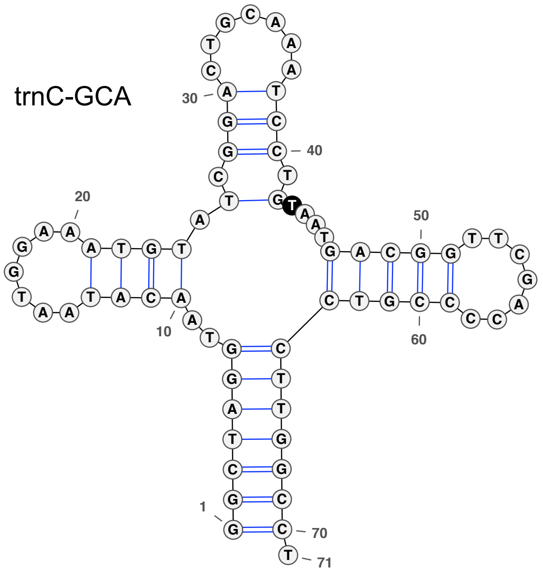

trnE-TTC

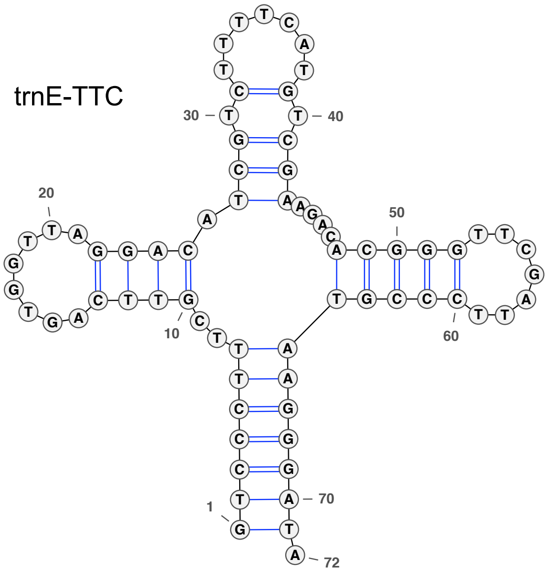

trnfM-CAT

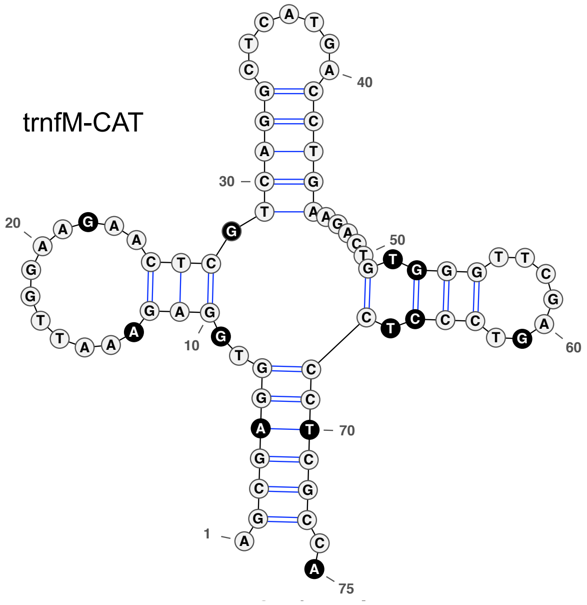

trnI-CAT

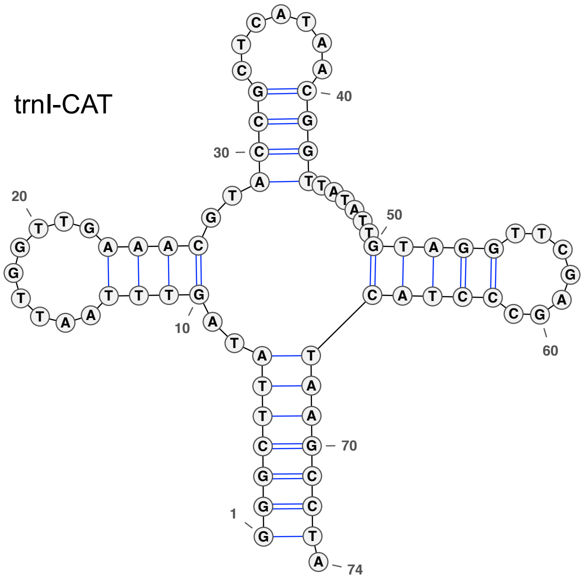

trnP-TGG

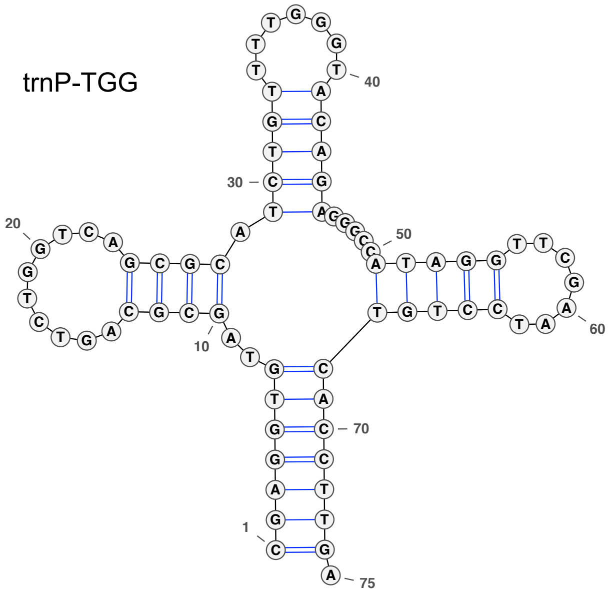

trnY-GTA

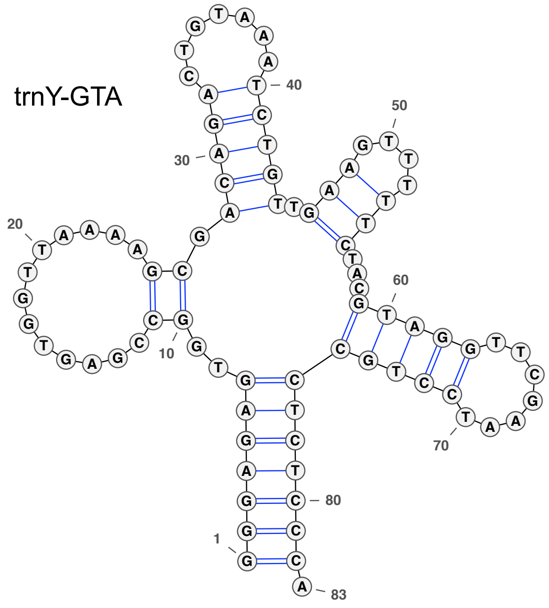

Chloroplast-like tRNAs

trnH-GTG

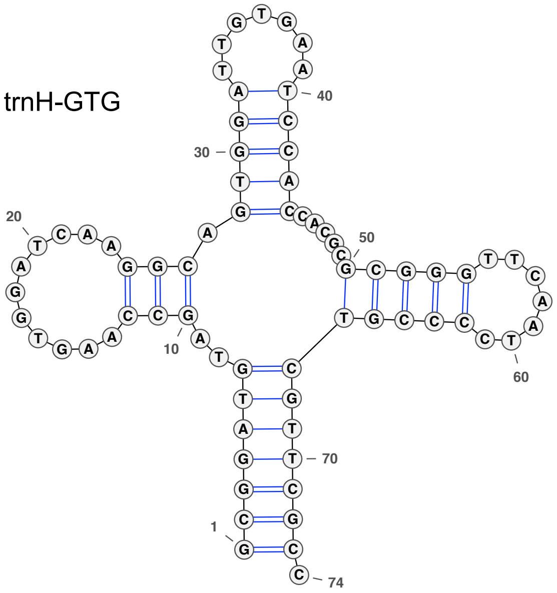

trnM-CAT

Possible pseudogene (anticodon not conserved)

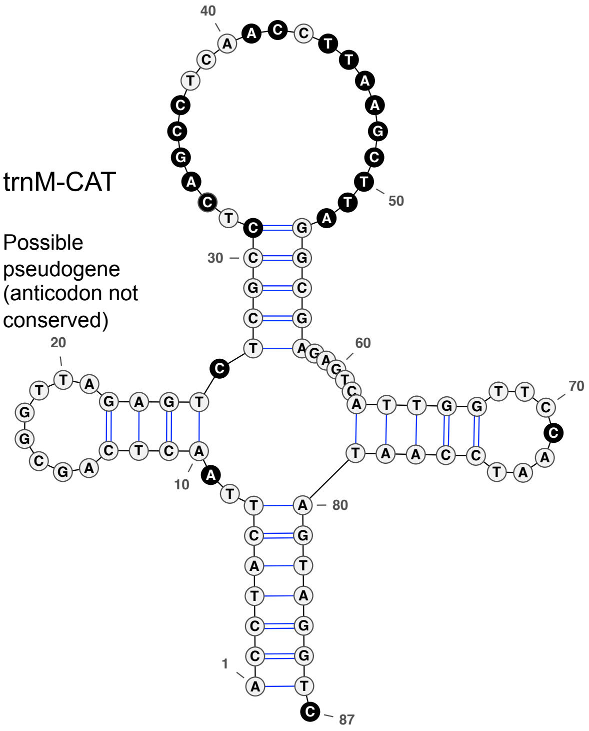

trnN-GTT

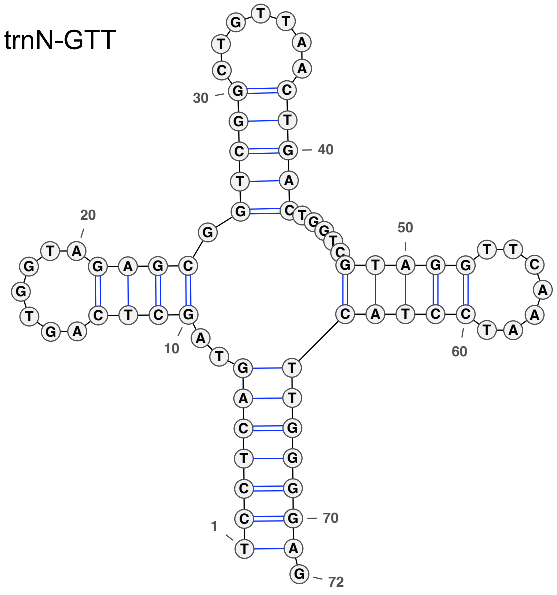

trnP-UGG

Possible pseudogene (anticodon not conserved)

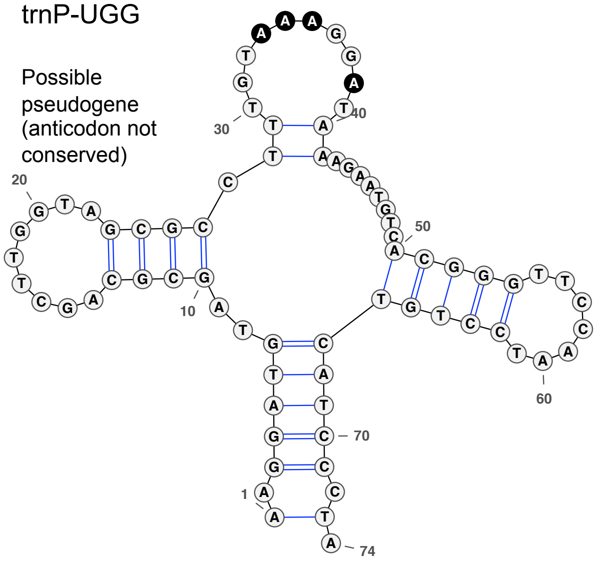

trnW-CCA

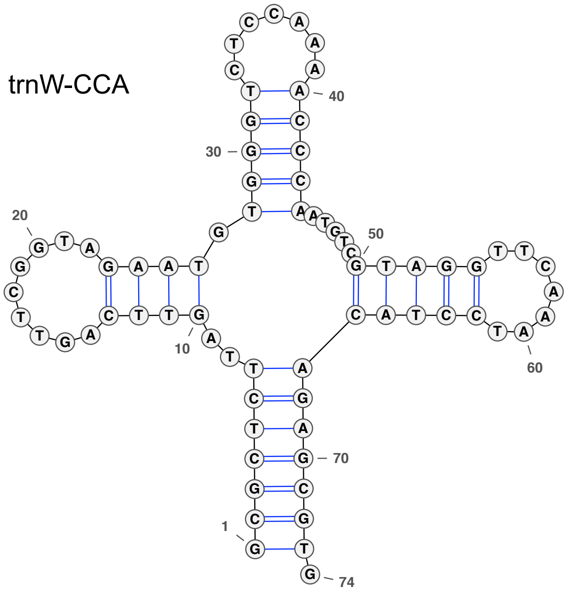

Additional File 2. Secondary structures for the 11 tRNA genes identified in the *Silene latifolia* mitochondrial genome. The structures were determined by tRNAscan-SE and drawn with VARNA. Black circles indicate sites that have experienced a substitution or an insertion in the *S. latifolia* lineage since its divergence from *Beta*.
