# Supplementary material for: Extensive loss of translational genes in the structurally dynamic mitochondrial genome of the angiosperm Silene latifolia
Source: BMC Evol Biol. 2010 Sep 10;10:274. doi: 10.1186/1471-2148-10-274 (PMC2942850; doi:10.1186/1471-2148-10-274)
Supplement: Additional file 3 — Summary of codon usage. [file 1471-2148-10-274-S3.PDF]

Additional File 3. Relative synonymous codon usage in the mitochondrial genomes of *Silene latifolia* and *Beta vulgaris*

		Count		RSCU	
	**Codon**	***Silene***	***Beta***	***Silene***	***Beta***
**Ala**	**GCA**	89	92	0.94	0.98
	**GCC**	79	78	0.84	0.83
	**GCG**	37	37	0.39	0.39
	**GCT**	172	170	1.82	1.80
**Arg**	**AGA**	44	44	1.28	1.25
	**AGG**	17	17	0.49	0.48
	**CGA**	54	58	1.57	1.64
	**CGC**	28	24	0.81	0.68
	**CGG**	19	20	0.55	0.57
	**CGT**	45	49	1.30	1.39
**Asn**	**AAC**	36	35	0.49	0.47
	**AAT**	112	113	1.51	1.53
**Asp**	**GAC**	40	39	0.52	0.51
	**GAT**	114	114	1.48	1.49
**Cys**	**TGC**	21	21	0.64	0.63
	**TGT**	45	46	1.36	1.37
**Gln**	**CAA**	106	104	1.59	1.60
	**CAG**	27	26	0.41	0.40
**Glu**	**GAA**	124	125	1.42	1.41
	**GAG**	51	52	0.58	0.59
**Gly**	**GGA**	137	138	1.51	1.52
	**GGC**	47	41	0.52	0.45
	**GGG**	56	57	0.62	0.63
	**GGT**	124	128	1.36	1.41
**His**	**CAC**	16	15	0.30	0.28
	**CAT**	89	91	1.70	1.72
**Ile**	**ATA**	106	103	0.74	0.72
	**ATC**	101	102	0.70	0.72
	**ATT**	225	222	1.56	1.56

		Count		RSCU	
	**Codon**	***Silene***	***Beta***	***Silene***	***Beta***
**Leu**	**CTA**	95	95	0.93	0.94
	**CTC**	54	52	0.53	0.51
	**CTG**	36	40	0.35	0.39
	**CTT**	126	128	1.23	1.26
	**TTA**	194	181	1.90	1.79
	**TTG**	108	112	1.06	1.11
**Lys**	**AAA**	93	93	1.29	1.31
	**AAG**	51	49	0.71	0.69
**Phe**	**TTC**	152	150	0.74	0.74
	**TTT**	260	255	1.26	1.26
**Pro**	**CCA**	71	74	1.17	1.20
	**CCC**	45	51	0.74	0.83
	**CCG**	34	33	0.56	0.53
	**CCT**	92	89	1.52	1.44
**Ser**	**AGC**	41	41	0.58	0.57
	**AGT**	98	95	1.39	1.33
	**TCA**	83	83	1.17	1.16
	**TCC**	46	53	0.65	0.74
	**TCG**	42	45	0.59	0.63
	**TCT**	114	111	1.61	1.56
**Thr**	**ACA**	60	61	0.90	0.91
	**ACC**	71	73	1.06	1.09
	**ACG**	28	31	0.42	0.46
	**ACT**	108	103	1.62	1.54
**Tyr**	**TAC**	31	33	0.34	0.36
	**TAT**	150	148	1.66	1.64
**Val**	**GTA**	110	116	1.19	1.25
	**GTC**	61	59	0.66	0.64
	**GTG**	77	74	0.83	0.80
	**GTT**	122	122	1.32	1.32

Codons highlighted in gray are those for which the corresponding tRNA gene appears to have been lost from the *S. latifolia* mitochondrial genome since its divergence from *Beta.* RSCU or relative synonymous codon usage is a statistical measure of whether a specific codon is preferentially used in protein genes (Sharp et al. 1986 Nucleic Acids Res. 14:5125–5143). Values greater than 1 indicate that the codon is overrepresented relative to the other codons for the same amino acid. Conversely, values below 1 indicate that the codon is underrepresented. Note that these data are based on genomic sequences and do not reflect changes resulting from RNA editing. Codon usage statistics were calculated with codonW v1.4.4.
